# New Mouse Hippocampal Cells Born in Pups and Adults Function Similarly

**DOI:** 10.1371/journal.pbio.0040441

**Published:** 2006-11-21

**Authors:** Mary Hoff

For the most part, the production of new brain cells in mammals occurs only in immature individuals at the time the nervous system is undergoing development. One notable exception is the hippocampus, a part of the brain involved in memory and spatial perception. Hippocampal cells called dentate granule cells (DGCs) develop in adults as well as in young animals. How these “adult-born” cells build their connections with the rest of the brain, and the extent to which they resemble “pup-born” cells, is of great interest to those who would like to coax other parts of adult brains to make new cells as a strategy for reversing the loss of function from trauma or degenerative disorders.

The development of DGCs is linked to hippocampus-related learning and behavioral changes. Developing, adult-born DGCs have different properties than mature neurons that arose when the brain was developing. Do the adult-born cells keep their distinct traits, or do they become functionally similar to the cells that developed early in life? To find out, Diego Laplagne, Alejandro Schinder, and colleagues compared the structure and function of these “new kids on the block” with DGCs that developed in the perinatal period in mice.

The researchers’ first task was to figure out a way to distinguish between pup-born and adult-born DGCs in brain tissue that contained both. To accomplish that task, they used retroviruses to introduce one kind of fluorescent protein into the developing DGCs at 7 days after birth and a second protein into the adult mouse brain at 42 days after birth. As a result of this treatment, the pup-born cells fluoresced green and the adult-born cells fluoresced red, making them readily distinguishable in brain slices.

Once they could tell the two types of cells apart, the researchers began testing a variety of electrophysiological traits of the connections between DGCs and neurons providing excitatory and inhibitory inputs. Using brain slices obtained from 19-week-old mice that had undergone the retrovirus labeling earlier in life, they looked at glutamatergic (excitatory) nerves connecting the hippocampus with the entorhinal cortex, another brain area associated with memory. When they stimulated the afferent excitatory neurons (which carry information from the neocortex to the hippocampus), the researchers evoked similar excitatory postsynaptic currents (EPSCs) in both pup-born and adult-born DGCs. When given paired-pulse stimulation, in which two pulses of electricity are given close together, the two types of cells showed a very similar reduction in EPSCs with the second pulse, suggesting that the short-term plasticity of the synapses is identical. And when the cells were given high-frequency stimulation, EPSC amplitude was depressed in both cell types in a similar manner. Thus, the researchers concluded that excitatory inputs to pup-born and adult-born DGCs are functionally similar.

**Figure:**
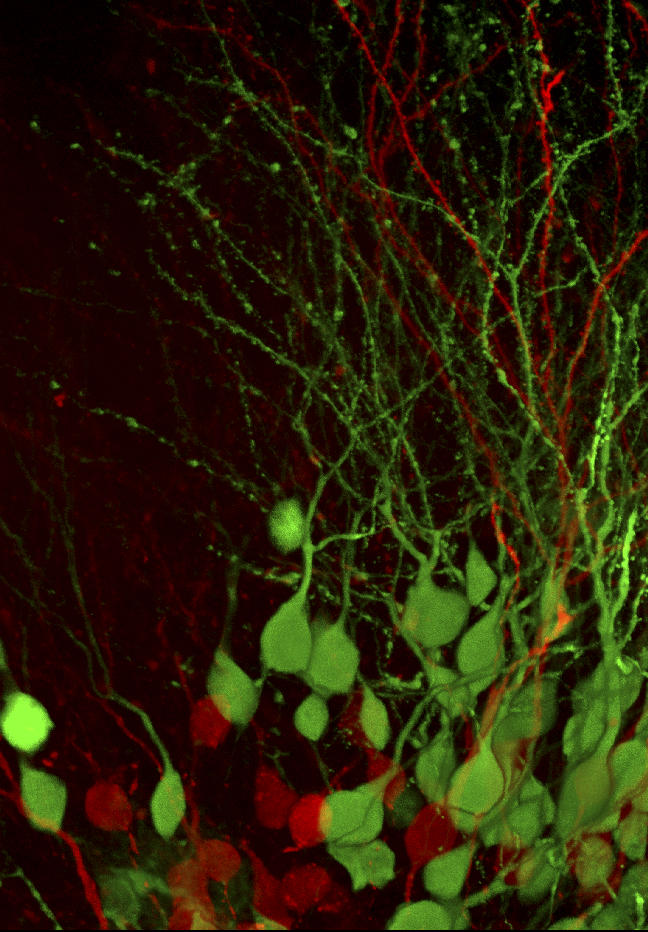
Dentate granule cells generated in the developing and adult hippocampus retrovirally labeled with green and red fluorescent proteins.

Next the researchers looked at GABAergic (inhibitory) inputs from interneurons that connect to the body and dendrites of the DGCs. Using brain slices from 14-week-old mice, they stimulated the incoming inhibitory neurons from the granule cell layer (GCL) and molecular layer (ML) of the hippocampus and found no significant difference in the amplitude and kinetics of inhibitory postsynaptic currents (IPSCs) that resulted. Spontaneous IPSCs, which add information about GABAergic inputs from areas other than the GCL and ML, exhibited similar frequency, amplitude, and kinetics in the pup-born and adult-born cells.

Having shown that pup-born and adult-born DGCs respond to both excitatory and inhibitory inputs in the same way, the researchers next turned their attention to how the two types of cells integrate the signals from the various inputs to produce an action potential (which transmits the signal), or spike. Spiking probability varied among neurons but was not distinguishable between the two cell types, further supporting the earlier indications that adult-born and pup-born DGCs function in fundamentally the same way.

With glutamatergic and GABAergic inputs handled similarly and the action-potential response indistinguishable, the researchers concluded that mature adult-born and pup-born DGCs are functionally similar. This means that at least some neurons that develop in adult brains can form connections that are indistinguishable from connections formed by neurons that develop early in life—a hopeful finding for those who have set their sights on one day being able to repair damaged or deteriorated brain tissue.

